# Effect of a labor triage checklist and ultrasound on obstetric referral at three primary health centers in Eastern Uganda

**DOI:** 10.1002/ijgo.13420

**Published:** 2020-11-04

**Authors:** Nicole Santos, Jude Mulowooza, Nathan Isabirye, Innocent Inhensiko, Nancy L. Sloan, Sachita Shah, Elizabeth Butrick, Peter Waiswa, Dilys Walker

**Affiliations:** ^1^ Institute for Global Health Sciences University of California San Francisco San Francisco CA USA; ^2^ School of Public Health Makerere University Kampala Uganda; ^3^ Department of Emergency Medicine University of Washington Seattle WA USA; ^4^ Global Health Department of Public Health Sciences Karolinska Institutet Stockholm Sweden; ^5^ Department of Obstetrics, Gynaecology and Reproductive Sciences University of California San Francisco San Francisco CA USA

**Keywords:** Checklist, Maternity triage, Primary health center, Referral, Uganda, Ultrasound

## Abstract

**Objective:**

To test whether introduction of a midwife‐performed triage checklist and focused ultrasound improves diagnosis and referral for obstetric conditions, including multiple gestation, placenta previa, oligohydramnios, preterm birth, malpresentation, and abnormal fetal heart rate.

**Methods:**

We implemented an intake log (Phase 1), a checklist (Phase 2), and a checklist plus ultrasound scan (Phase 3) at three primary health centers in Eastern Uganda for women presenting in labor. Intake diagnoses, referral status, and delivery outcomes were assessed, as well as sensitivity and positive predictive value (PPV).

**Results:**

Between February 2018 and July 2019, 1155, 961, and 603 women were enrolled across the three phases (n=2719); 2339 had outcome data. Incidence of any outcome‐confirmed condition was 8.8%, 7.9%, and 7.1% (*P*=0.526) for each phase, respectively. The proportion of referred women with a condition did not change between Phases 1 and 2 (7.8% versus 8.6%, *P*=0.855), but increased in Phase 3 (48.4%, *P*<0.001). Sensitivity improved with each intervention; PPV decreased with ultrasound.

**Conclusion:**

Use of ultrasound plus checklist increased referrals and sensitivity for high‐risk conditions, with decreased PPV. The checklist alone improved correct diagnosis, but not referral. Further evaluation of these triage interventions to maximize diagnostic accuracy, referral decisions, and outcomes are warranted.

## Abstract 2

Triage ultrasound increased referrals for both correct and incorrect diagnosis of six obstetric conditions, whereas a triage checklist improved correct identification but not referral.

## 1 INTRODUCTION

Nearly three‐quarters of the estimated 295 000 annual maternal deaths are due to direct obstetric complications and half of 2.6 million third‐trimester stillbirths occur during labor and delivery.^1^, ^2^ Improved quality of care, particularly during the intrapartum period, can reduce preventable maternal and perinatal mortality. In many low‐ and middle‐income countries, failure to receive adequate care when a facility is reached, the third delay,^3^ is exacerbated by lack of supplies, personnel shortages, long waiting times, and weak referral protocols.^4^

Effective referral from primary health centers (PHCs) to hospitals offering comprehensive emergency obstetric and neonatal care is a critical component of quality maternity care.^5^ Inter‐facility referral relies on a confluence of factors, such as timely arrival of a woman in labor, appropriate identification of high‐risk conditions by providers, emergency transportation, and communication between the referring and receiving facilities.^6^ However, inconsistent understanding of clinical criteria for referral, guideline non‐compliance, inadequate clinical skills, lack of confidence in decision‐making, and absence of transportation remain critical gaps at PHCs.^7^, ^8^, ^9^ These barriers are exacerbated by weak communication across the health system, and further delays when a referral hospital is reached.

Labor triage, when a woman first presents to the PHC maternity unit in labor, is an opportunity to screen for conditions that may warrant referral, especially in contexts where prenatal care screening is poor. Although the WHO Safe Childbirth Checklist begins with "Does mother need referral?", standardized assessments before decision to admit, refer, or send home are not included.^10^ Triage practices at higher‐level referral facilities in resource constrained settings have been explored, such as implementation of a Traffic Light System, interactive training programs, and standardized documentation^11^, ^12^; however, these interventions have not been introduced at the PHC level.

This study aimed to evaluate if triage interventions—a checklist, focused ultrasound scan and referral transportation support—improved the ability of PHC midwives to correctly diagnose high‐risk conditions and appropriately initiate referral to the district hospital (DH).

## 2 MATERIALS AND METHODS

We examined the phased implementation of triage interventions at three PHCs in Busoga region, Uganda, between February 2018 and July 2019. In 2016, 60% of Ugandan women received four prenatal care visits and the median length of pregnancy at entry to prenatal care was 4.7 months.^13^

The three study PHCs provide 24‐hour delivery services without cesarean delivery capacity, conduct 60–75 monthly deliveries, and are located 11, 25, and 41 km from the DH. On average, each PHC has five midwives on staff with each shift covered by one midwife in the labor room and another in prenatal care. The standard of care guidance is to refer to higher care for the conditions of interest (detailed below) unless delivery is imminent. Other conditions including obstructed/prolonged labor, previous cesarean section, pre‐eclamptic toxemia, and antepartum hemorrhage, also warrant referral. Ambulances are accessible, but patients pay money for fuel or rely on their own means to reach the DH. Ultrasounds were not available before the study.

The study’s primary outcome was the proportion of women with one or more of six high‐risk conditions confirmed at birth who were referred upon initial PHC presentation. The conditions (preterm birth, multiple gestation, oligohydramnios, placenta previa, malpresentation, and abnormal fetal heart rate) were combined into one composite variable for the primary analysis. The following criteria were used to confirm presence of a complication at outcome: multiple gestation, more than one fetus present; preterm birth, gestational age by Ballard examination; oligohydramnios, reduced amniotic fluid at birth without rupture of membranes; placenta previa, if reported by vaginal examination or cesarean section; malpresentation, non‐cephalic presenting fetal part; abnormal fetal heart rate, 1‐minute Apgar scores less than 7 or infant born without signs of life.

The study interventions are described in Table 1. The study evaluated the effect of Phase 2 and Phase 3 interventions on the primary outcome using Phase 1 as the baseline comparison. Phase 1 introduced a triage intake log and outcome form. In Phase 2, standardized documentation was supplemented with a triage checklist and referral support. Ultrasound (Mindray DP‐10, Mindray, Shenzhen, China) was added in Phase 3. Phase 2 and Phase 3 checklists are provided in the Appendix S1. Documentation, checklist, and ultrasound were also introduced at the referral DH, as part of a concurrent study that will be described elsewhere. The ultrasound curriulum and quality assurance activites are published elsewhere.^14^

**Table 1:** Description of triage interventions introduced at the three primary health centers (PHCs) during each study phase.

Intervention description		How it was implemented	Phases
Standardized documentation at intake and outcome	**Intake log:** documents a single line for entry of each patient assessed in triage, including demographic information, gestational age at presentation, and clinical diagnoses	Midwife assessed each woman before deciding to admit, send home, or refer	1,2,3
	**Outcome form**: records diagnoses confirmed at outcome	Midwife confirmed and documented delivery outcomes in study tools, medical chart, and maternity register	1,2,3
Clinical interventions^a^	**Triage checklist:** prompts providers to perform clinical assessments (e.g., vital signs, gestational age, head position) and guides to appropriate management, including referral	Before filling out the intake log, midwife evaluated each woman using this checklist to standardize clinical assessments	2,3
	**Focused ultrasound scan:** assesses fetal cardiac activity, number of fetuses, head position, placental location, amniotic fluid volume, and fetal biometry measures (head circumference, biparietal diameter, femur length)	Before filling out the intake log and after using the Phase 2 clinical checklist, midwife assessed each woman by focused ultrasound	3
Referral support	**Ambulance fuel reimbursement plus driver and midwife allowance:** supports transport of the mother, birth companion if any, and an escorting midwife if available to the DH	Provided by the research study	2,3
	**Airtime:** supports communication between PHC and DH midwives regarding referrals	Provided by the research study	2,3

^a^ The checklists used in Phases 2 and 3 are provided in Appendix S1.

Women who presented with labor‐like pains after 28 weeks of pregnancy were eligible. Women were excluded if they were not in labor or required immediate intervention, such as those with severe antepartum hemorrhage, eclamptic seizure, or imminent delivery.

We designed a balanced study with an equal number of women per phase. We estimated that 4% of all parturient women were referred for one of the six conditions based on baseline assessment of register data. Given a two‐tailed test, α of 0.05, 80% power, and a relative effect of 100% (from 4% to 8%), the study required 601 women across the three PHCs per phase (Fleiss continuity correction applied). The sample size was increased by 20%, to 721 per phase, to account for loss to follow up, refusal, and missing data.

For each phase, three midwives and one study research nurse from each PHC were trained in study procedures. One study‐trained midwife covered each shift with support from the research nurse. Tools were piloted and revised before implementation.

Study‐trained midwives filled out paper‐based study tools and related clinical data sources (i.e., medical charts, register). Research nurses identified eligible women, obtained informed written consent, and entered data using tablets into open data kit. They verified data completeness and consistency before entering data electronically. The study data manager performed biweekly data quality spot‐checks, transferred data to a secure server, and obtained monthly counts of admissions and deliveries to estimate enrollment rates.

Paper forms were kept in secure cabinets. All devices were encrypted and password protected, and all electronic data were kept on secure systems. Data access was limited to designated study staff, including the open data kit server, which was hosted by University of California San Francisco.

SPSS v25.0 (IBM, Armonk, NY, USA) was used to conduct range and logic checks, and to clean and analyze the data. Individual‐level data from the intake log, outcome form, and Phase 2 and Phase 3 checklists were linked by unique study identification numbers and inpatient numbers. Bivariate analyses included χ^2^ tests or Fisher’s exact statistics for categorical data and Student’s *t* tests for continuous data.

Conditions were examined using composite variables. Any maternal condition comprises conditions that were measured once per pregnancy (multiple gestation, preterm birth, oligohydramnios, placenta previa). Any fetal condition comprises conditions that were measured per fetus for both singleton and multiple gestations (malpresentation, abnormal fetal heart rate). Any maternal or fetal condition is a composite variable for the six conditions used for the primary analysis. Data for individual conditions are presented without *P* values to avoid over‐interpretation. Logistic regression was used to adjust for covariates.

We ascertained sensitivity and positive predictive value (PPV), as well as specificity and negative predictive value. As secondary outcomes, without a priori hypotheses, descriptive analyses without multiple comparison adjustments were conducted.

All participants provided voluntary written informed consent. Approvals were obtained from University of California San Francisco’s Institutional Review Board (#17‐23310) and the Higher Degrees, Research and Ethics Committee at Makerere University (#515).

## 3 RESULTS

**FIGURE 1:**
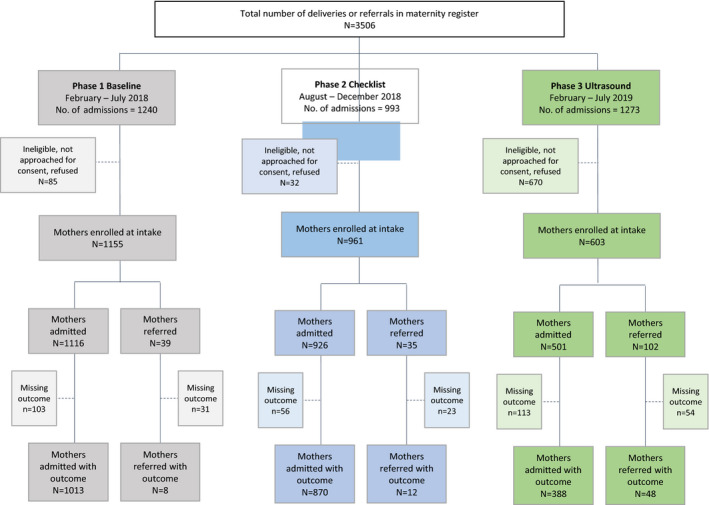
Study flow diagram by phase.

In Phases 1, 2, and 3, 93.1% (1155), 96.8% (961), and 47.4% (603) of admissions were enrolled (total n=2719, Fig. 1). Most women were less than 35 years old (24.2 ± 5.6) and had completed some primary level education. The average gestational weeks at intake was similar across phases (38.3 ± 1.9) (Table 2). Overall referral rates for any reason increased across the phases: 3.4% (39/1155), 3.6% (35/961), and 16.9% (102/603).

**Table 2:** Demographic characteristics of study participants by phase (N=2719), collected at intake.

	Phase 1 (n=1155)		Phase 2 (n=961)		Phase 3 (n=603)		*P* value	
	n	%	n	%	n	%	Phase 2 vs Phase 1	Phase 3 vs Phase 1
Maternal age								
<20 years	255	22.1%	218	22.7%	156	26.0%	0.484	0.182
20–35 years	829	71.9%	674	70.1%	408	67.9%		
>35 years	69	6.0%	69	7.2%	37	6.2%		
Mean (SD)	24.2 (5.44)		24.4 (5.8)		23.8 (5.6)		0.424	0.110
Education level								
None	16	1.4%	11	1.1%	2	0.3%	0.174	0.031
Some/completed primary	794	69.0%	696	72.4%	440	73.5%		
Some/completed secondary	322	28.0%	246	25.6%	143	23.9%		
Some/completed university	19	1.7%	8	0.8%	14	2.3%		
Gestational age at intake, mean (SD)	38.3 (2.04)		38.3 (1.67)		38.6 (2.02)		0.798	0.002

Outcome data were obtained on 2339 deliveries, including women who were admitted to the PHC upon presentation (n=2271), or women referred to the DH or re‐admitted as the result of failed referral (n=68). The proportion of PHC‐admitted women with known outcomes was 90.8% in Phase 1, 94.0% in Phase 2, and 77.4% in Phase 3. The proportion of completed or failed referrals with known outcomes was 20.5% in Phase 1, 34.2% in Phase 2, and 47.1% in Phase 3 (Fig. 1). The achieved harmonic mean sample size was 946 for Phase 1 versus Phase 2 comparisons and 611 for Phase 1 versus Phase 3 comparisons, both above the required balanced sample size of 601.

The incidence of any maternal or fetal condition, as defined by post‐delivery outcomes, was 8.8%, 7.9%, and 7.1% across phases (*P*=0.526, Table 3). Preterm birth, malpresentation, and abnormal fetal heart rate were most common. We observed no differences in adverse maternal or newborn outcomes between phases though the numbers were small and the study was not powered to assess this (Table S1).

**Table 3:** Maternal and fetal complications incidence, as defined by outcome diagnosis (N=2339)

Condition confirmed at outcome	Phase 1		Phase 2		Phase 3		*P* value	
	n	%	n	%	n	%	Phase 2 vs Phase 1	Phase 3 vs Phase1
Multiple gestation	4	0.4%	10	1.1%	4	0.9%	0.059	0.250^a^
Oligohydramnios	9	0.9%	8	0.9%	2	0.5%	0.953	0.522^a^
Placenta previa	2	0.2%	1	0.1%	1	0.2%	1.000^a^	1.000^a^
Preterm birth	53	5.2%	33	3.7%	15	3.4%	0.129	0.147
Malpresentation	23	2.3%	10	1.1%	6	1.4%	0.062	0.273
Abnormal fetal heart rate	8	0.8%	20	2.3%	10	2.3%	0.007	0.017
Maternal condition	66	6.5%	49	5.6%	19	4.4%	0.407	0.116
Fetal condition	30	2.9%	28	3.2%	16	3.7%	0.765	0.465
Any maternal or fetal condition	90	8.8%	70	7.9%	31	7.1%	0.491	0.280

^a^ Fisher’s exact test.

**FIGURE 2:**
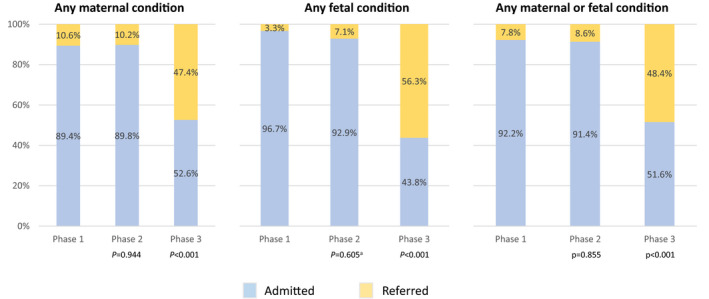
Maternal disposition at intake among those with an outcome‐defined condition (n=2339). Conditions were examined by composite variables: any maternal condition (multiple gestation, preterm birth, oligohydramnios, placenta previa); any fetal condition (malpresentation, abnormal fetal heart rate); any maternal or fetal condition for the primary analysis. Referred includes those who completed referral to the DH and those who were re‐admitted to the PHC after failed referral. *P* values presented compare Phase 1 versus Phase 2 and Phase 1 versus Phase 3. ^a^Fisher’s exact test.

Among women who had any outcome‐confirmed maternal or fetal condition, the proportion who were referred or re‐admitted as the result of failed referral in Phases 1 and 2 were similar (7.8% versus 8.6%, *P*=0.855, Fig. 2), while Phase 3 referral increased significantly (48.4%, *P*<0.001). Similar trends were observed for any maternal or any fetal condition. Results for these three composite outcomes were consistent when controlled for maternal age, education level, fuel source, attendance to four prenatal care visits, gestational age at intake, nulliparity, history of cesarean, as well as phase‐specific differences in the incidence of any individual condition (data not shown). Though only powered to assess the composite variable, referral increased in Phase 3 for women who had outcome‐confirmed preterm birth, malpresentation, and abnormal fetal heart rate (Table S2).

Referral rates for only completed DH referrals were 2.2%, 4.3%, and 35.5% (*P*<0.001). Among enrolled women with an intake diagnosis of one or more of the conditions of interest (Table S3), intent to refer similarly increased between Phases 1 and 3 (14.4% versus 41.3%, *P*<0.001), but not between Phases 1 and 2 (14.4% versus 17.7%).

Comparing Phase 1 with Phase 2, the checklist increased diagnostic sensitivity for any maternal condition (Table 4; Phase 1 57.6%, Phase 2 65.3%, *P*=0.401), any fetal condition (10% versus 57.1%, *P*<0.001), and any maternal or fetal condition (41.1% versus 60%, *P*=0.018). Phase 2 PPV for the three composite variables trended upward non‐significantly. Comparing Phases 1 and 3, the checklist plus scan substantially increased sensitivity for all composite variables (not significant for maternal conditions). Phase 3 PPV significantly decreased for any maternal condition (51.3% versus 26.3%, *P*=0.004) and any maternal or fetal condition (49.4% versus 32.4%, *P*=0.032) compared with Phase 1, but was not significantly higher for the limited number of any fetal conditions. Specificity and negative predictive value are presented in the Table S4. Comparing Phase 1 with Phase 3, the checklist plus scan decreased specificity for any maternal conditions and the composite outcome, indicating increased false positives, as was reflected in changes in PPV.

**Table 4:** Sensitivity and positive predictive value for conditions of interest among all women who have an outcome (N=2339), inclusive of those admitted/delivered at the primary health center (n=2271) and those referred and have outcome (n=68).

	Phase 1		Phase 2		Phase 3		p‐value	
	n_1_	%	n_1_	%	n_1_	%	Phase 2 vs Phase 1	Phase 3 vs Phase 1
Sensitivity								
Multiple gestation	2	50.0%	5	50.0%	3	75.0%		
Oligohydramnios	3	33.3%	4	50.0%	0	0.0%		
Placenta previa	0	0.0%	0	0.0%	0	0.0%		
Preterm birth	35	66.0%	25	75.8%	14	93.3%		
Malpresentation	4	17.4%	7	70.0%	4	66.7%		
Abnormal fetal heart rate	0	0.0%	11	55.0%	6	60.0%		
Maternal condition	38	57.6%	32	65.3%	14	73.7%	0.401	0.204
Fetal condition	3	10.0%	16	57.1%	10	62.5%	<0.001^a^	<0.001^a^
Any maternal or fetal condition	37	41.1%	42	60.0%	21	67.7%	0.018	0.010
Positive predictive value								
Multiple gestation	2	33.3%	5	62.5%	3	100.0%		
Oligohydramnios	3	100.0%	4	80.0%	0	0.0%		
Placenta previa	0	0.0%	0	0.0%	0	0.0%		
Preterm birth	35	49.3%	25	56.8%	14	29.2%		
Malpresentation	4	50.0%	7	70.0%	4	100.0%		
Abnormal fetal heart rate	0	0.0%	11	73.3%	6	46.2%		
Maternal condition	40	51.3%	32	60.4%	15	26.3%	0.304	0.004
Fetal condition	4	36.4%	16	69.6%	10	58.8%	0.135^a^	0.440^a^
Any maternal or fetal condition	42	49.4%	45	63.4%	23	32.4%	0.080	0.032

n1 = # true positives; denominator includes false negatives; n2 = # true positives; denominator includes false positives

^a^ Fisher’s exact test.

## 4 DISCUSSION

Phase 3 interventions (checklist plus ultrasound, referral support) increased appropriate referral for the outcome‐confirmed conditions of interest. Although Phase 1 referral rates were nearly double those originally estimated (7.8% versus 4%), the substantial increase to 48.4% indicates that ultrasound increased diagnostic suspicion and the decision to refer for these conditions. Ultrasound increased sensitivity for the composite outcome, but it decreased PPV and specificity. Hence, there was an increased incidence of false‐positive diagnoses, as is common with increasing detection. Increased referral of true positives is essential, but has the drawback of increasing burden of care for false positives referred, particularly when system resources at either the PHC or the higher level facility are limited.

The impact of ultrasound on referral has been previously explored in the prenatal care context. The First Look Study, which introduced routine ultrasound across PHCs in five low‐ and middle‐income countries, did not increase delivery of complicated cases in hospitals with cesarean capacity.^15^ Critical factors that limited successful referral were cost, transportation, and distance, which were beyond the scope of their study.^16^ Our study ameliorated some of these barriers by supporting fuel reimbursement and facilitating communication at no cost to the PHC or to the woman, though important considerations like road conditions and distance remained.^17^ Referral support could have increased intent and ability to refer, but unlike Phase 3, Phase 2 did not change overall referral rates, reinforcing the catalytic role of ultrasound in clinical decision‐making to refer. This finding is consistent with the Better Birth trial, where implementation of the WHO Safe Childbirth Checklist in India showed no differences in inter‐facility referral between intervention and control groups.^10^

Unlike checklist plus ultrasound, however, the Phase 2 checklist increased sensitivity without changing PPV or specificity, suggesting increased correct identification (i.e., more true positives without more false positives). Standardized checklists and locally tailored guidelines have demonstrated improved outcomes and uptake of evidence‐based practices, such as a recent labor management intervention in Tanzania^18^ and the WHO Safe Childbirth Checklist,^10^ respectively. Hence, checklists may be useful cognitive aids to standardize triage practices.^19^

Although our findings suggest that focused, midwife‐performed ultrasound at PHC triage can increase appropriate inter‐facility referral for complicated cases, several limitations exist. First, follow up among those referred varied across phases, reducing complete assessment of the primary outcome, appropriate referral for correctly identified conditions. As the number of referrals with known outcomes was smaller in Phase 1 than subsequent phases, the baseline rate could have been higher. Moreover, for Phases 2 and 3, if condition‐related referrals with missed outcome had the condition confirmed (true positive), the effect would be larger; conversely, incorrect diagnosis at outcome (false positive) would have decreased the effect. We attempted to link referrals from the PHCs to the DH and identify final diagnoses from maternity registers, but the data were not collected in the registers in a standardized manner. Additionally, many referred women self‐reported going to private facilities or traditional birth attendants, which hindered follow up and acquisition of provider‐confirmed delivery data. Improved capacity for follow up within the health system is needed to better understand maternal and perinatal mortality and morbidity data, and opportunities for intervention.

Other study limitations include potential suspicion bias, given that the midwives identified both intake and outcome diagnoses, and inaccuracy of the outcome diagnoses used to determine sensitivity and PPV. Under‐reporting of the conditions at outcome, whether due to misdiagnosis or data quality, could have compromised the study results. To this end, we conducted post‐hoc analyses excluding oligohydramnios from the primary and secondary outcomes, given the subjective nature of oligohydramnios assessment post‐delivery without the use of ultrasound,^20^, ^21^ as well as the inconsistent assessment or documentation of premature rupture of membranes. Trends in referral remained the same upon exclusion of oligohydramnios (Table S5A), but Phase 3 PPV was no longer significantly lower for any maternal or fetal condition compared to Phase 1 when oligohydramnios was excluded (Table S5B). The latter finding suggests that false‐positive diagnosis of oligohydramnios contributed to the initial results of decreased PPV for the composite variable.

Additionally, several implementation challenges impeded ultrasound use. Small delivery volumes and mothers who arrived in second‐stage labor reduced implementation and practice, leading to enrollment delays. Best practice for ultrasound training involves theoretical teaching directly followed by high volume of proctored practice scans to cement key concepts and physical skills.^22^ The study‐trained midwives needed adequate hands‐on practice to sufficiently pass their observed structured clinical examinations before enrolling; only half were enrolling 2 months into Phase 3. Staff turnover and significant electricity outages, sometimes for multiple days, impeded ultrasound use. Collectively, these factors contributed to lower Phase 3 enrollment, though the required harmonic mean sample was achieved. Other ultrasound‐related factors may have also introduced selection bias. As scans could take up to 30 minutes, midwives may have selectively scanned higher‐risk women, or those who had more positive health‐seeking behaviors, such as coming earlier in labor. Those missed during Phase 3 recruitment may have included lower‐risk deliveries, and so women in Phase 3 may be less representative than those in Phases 1 and 2. However, analyses controlled for confounding found similar results, reflecting internal validity, for the primary outcome.

Nonetheless, through a phased intervention approach, we assessed multiple triage interventions with robust statistical methods. Logistic regression analyses adjusted for phase differences to minimize the design limitation of a pre‐post study. We used a composite variable comprising several conditions of varying prevalence to assess the potential holistic benefit of these interventions. We also conservatively presented composite variables to avoid over‐interpretation of individual conditions’ results and secondary outcomes. It is worthwhile testing these interventions in other populations where prevalence of these conditions may vary.

Although this study solely evaluated PHC interventions that might improve referral, we concurrently implemented a similar study at the DH, which will yield important insights about the interventions’ utility in higher‐level care settings (manuscript submitted). Complementarity of interventions across the care continuum and health system tiers to improve identification of high‐risk obstetric conditions and referral is critical.

In conclusion, ultrasound use substantially increased referrals from PHCs to the DH for both correct and incorrect diagnoses of the six conditions. Its use in labor triage could benefit from more nuanced consideration of costs, facility resources, and implementation challenges. The PHC triage checklist and standardized documentation were less beneficial in terms of referral, but increased correct identification. Therefore, a future study examining the impact of both interventions on maternal and newborn outcomes is warranted, as well as evaluation of a more focused or targeted scan for select women at labor triage to maximize diagnostic accuracy and referral decisions.

## AUTHOR CONTRIBUTIONS

JM, NS, EB, and DW conceived the study, and NS, NLS, SS, and DW designed it. JM, NI, and II led in‐country implementation and data management in collaboration with PW, NS, SS, EB, and DW. SS led ultrasound‐related training activities. NS and NLS analyzed the data. NS, NLS, and JM contributed to manuscript composition and all authors helped to edit the final manuscript. Study funding was acquired by DW.

## CONFLICTS OF INTEREST

The authors of this study have no financial conflicts of interest to report.

## 5 DETAILS OF ETHICS APPROVAL

Ethics approval for this study was obtained from the Institutional Review Board at the University of California San Francisco IRB (#17‐23310) and the Higher Degrees, Research and Ethics Committee at Makerere University in Uganda (#515). Initial approvals were obtained October 28, 2017 and November 7, 2017, respectively, and renewed annually.

## Supporting information


**Table S1**. Maternal and newborn outcomes (n=2339).
**Table S2**. Maternal disposition at intake among those with an outcome‐defined condition (n=2339).
**Table S3**. Maternal disposition at intake among study population including those with or without outcome data (n=2719).
**Table S4**. Specificity and negative predictive value (NPV) for conditions of interest among all women who have an outcome (n=2339), inclusive of those admitted/delivered at the PHC (n=2271) and those referred and have outcome (n=68).
**Table S5**. (A) Maternal disposition at intake among those with an outcome‐defined condition (n=2339) excluding oligohydramnios from the composite variables. (B) Sensitivity and positive predictive value (PPV) for conditions of interest excluding oligohydramnios among all women who have an outcome (n=2339), inclusive of those admitted/delivered at the PHC (n=2271) and those referred and have outcome (n=68).
**Appendix S1**. Checklists for Phases 2 and 3.
